# The Nigerian Military Public Health Response to COVID-19: A 14-Month Appraisal

**DOI:** 10.1089/hs.2021.0143

**Published:** 2022-06-17

**Authors:** Ojor Ayemoba, Usman Adekanye, Michael Iroezindu, Ikenna Onoh, Ismail Lawal, Aminu Suleiman, Samuel Joshua, Amos Ogundeji, Yakubu Adamu, Dooshima Ugandem-Okonkwo, Funmilayo Owolabi, Mary Atang, Goodluck Nwagbara, Yaya Musa, Sunday Odeyemi, Abiodun Amosu, Ifeanyi Okoye, Yusuf Ahmed, Joshua Nalazai, Zubairu Elayo, Taiwo Adelanwa, Thomas Monday, Eddie Bloom, Emmanuel Benyeogor, Laura Chittenden, Nathan Okeji

**Affiliations:** Ojor Ayemoba, MBChB, FMCPath, is Clinical Research Advisor and Consultant Haematologist, Clinical Research, Ministry of Defence Health Implementation Programme, United States Army Medical Research Directorate – Africa/Nigeria, Abuja, Nigeria.; Usman Adekanye, MPH, is an Assistant Clinical Research Officer and Programme Veterinarian and Field Epidemiologist, Clinical Research, Ministry of Defence Health Implementation Programme, United States Army Medical Research Directorate – Africa/Nigeria, Abuja, Nigeria.; Samuel Joshua is Programme Lab Officer and Principal Laboratory Scientist, Clinical Laboratory Services, Ministry of Defence Health Implementation Programme, United States Army Medical Research Directorate – Africa/Nigeria, Abuja, Nigeria.; Funmilayo Owolabi is a Prevention Officer and Public Health Nurse, Preventive Health Services, Ministry of Defence Health Implementation Programme, United States Army Medical Research Directorate – Africa/Nigeria, Abuja, Nigeria.; Mary Atang, MSc, is a Training Officer and Public Health Nurse, Training, Ministry of Defence Health Implementation Programme, United States Army Medical Research Directorate – Africa/Nigeria, Abuja, Nigeria.; Goodluck Nwagbara, MSc, is Director and Chief Lab Scientist, Defence Reference Laboratory, Ministry of Defence Health Implementation Programme, United States Army Medical Research Directorate – Africa/Nigeria, Abuja, Nigeria.; Yaya Musa, MSc, is Acting/Ag Director and Chief Lab Scientist, Defence Reference Laboratory, Ministry of Defence Health Implementation Programme, United States Army Medical Research Directorate – Africa/Nigeria, Abuja, Nigeria.; Abiodun Amosu, MA, is Chief Medical Records Officer and Strategic Information Lead, Strategic Information, Ministry of Defence Health Implementation Programme, United States Army Medical Research Directorate – Africa/Nigeria, Abuja, Nigeria.; Joshua Nalazai, FPCPharm, is a Programme Pharmacist and Logistics Officer, Procurement and Logistics, Ministry of Defence Health Implementation Programme, United States Army Medical Research Directorate – Africa/Nigeria, Abuja, Nigeria.; Taiwo Adelanwa, PGD, is Deputy Director and a Public Health Nurse, Ministry of Defence Health Implementation Programme, United States Army Medical Research Directorate – Africa/Nigeria, Abuja, Nigeria.; Thomas Monday, MBA, PGD, is Assistant Director, Finance and Accounts, Ministry of Defence Health Implementation Programme, United States Army Medical Research Directorate – Africa/Nigeria, Abuja, Nigeria.; Nathan Okeji, MBBS, FWACS, is Director General and Consultant Obstetrician/Gynaecologist, Ministry of Defence Health Implementation Programme, United States Army Medical Research Directorate – Africa/Nigeria, Abuja, Nigeria.; Michael Iroezindu, FWACP, MPH, is Director of Research and Consultant Infectious Disease Physician, Clinical Research Center, United States Army Medical Research Directorate – Africa/Nigeria, Abuja, Nigeria.; Ismail Lawal, MPH, MBA, is Care and Treatment Lead and a Public Health Physician, Clinical Care and Treatment, United States Army Medical Research Directorate – Africa/Nigeria, Abuja, Nigeria.; Aminu Suleiman, PhD, MPH, is Laboratory Team Lead and Chief Medical Laboratory Scientist, Clinical Laboratory Services, United States Army Medical Research Directorate – Africa/Nigeria, Abuja, Nigeria.; Amos Ogundeji, MPH, PhD, is Lab TB-HIV Lead and Associate Director, Clinical Laboratory Services, United States Army Medical Research Directorate – Africa/Nigeria, Abuja, Nigeria.; Yakubu Adamu, FMCPH, is Deputy Director and a Public Health Physician, Public Health Programs and Policy, United States Army Medical Research Directorate – Africa/Nigeria, Abuja, Nigeria.; Dooshima Ugandem-Okonkwo, MSc, is Health Counselling Lead and Prevention Manager, Preventive Health Services, United States Army Medical Research Directorate – Africa/Nigeria, Abuja, Nigeria.; Sunday Odeyemi, MSc, is Associate Laboratory Director and Medical Laboratory Scientist, Defence Reference Laboratory, United States Army Medical Research Directorate – Africa/Nigeria, Abuja, Nigeria.; Ifeanyi Okoye, MInfSc, is Strategic Information Lead and a Public Health Physician, Strategic Information, United States Army Medical Research Directorate – Africa/Nigeria, Abuja, Nigeria.; Yusuf Ahmed, MPH, is Prevention Team Lead and a Public Health Physician, Preventive Health Services, United States Army Medical Research Directorate – Africa/Nigeria, Abuja, Nigeria.; Zubairu Elayo, MBA, is a Programme Logistician and Deputy Director, Procurement and Logistics, United States Army Medical Research Directorate – Africa/Nigeria, Abuja, Nigeria.; Eddie Bloom, MSc, MA, is Director, Administration and Operations, United States Army Medical Research Directorate – Africa/Nigeria, Abuja, Nigeria.; Laura Chittenden, PhD, is Country Director, United States Army Medical Research Directorate – Africa/Nigeria, Abuja, Nigeria.; Ikenna Onoh, FWACP, MSc, is a Research Fellow, Consultant Community Physician, and Field Epidemiologist, Health Emergency Preparedness and Response, Nigerian Field Epidemiology and Laboratory Training Programme, Abuja, Nigeria.; Emmanuel Benyeogor, MSc, is an Epidemiologist and Subnational PHEOC Coordination Assistant, Health Emergency Preparedness and Response, Nigeria Centre for Disease Control, Abuja, Nigeria.

**Keywords:** COVID-19, Epidemic management/response, Public health preparedness/response

## Abstract

The COVID-19 pandemic has caused significant morbidity and mortality since its emergence in December 2019. In Nigeria, the government inaugurated the Presidential Task Force on COVID-19 to coordinate resources while the Nigeria Centre for Disease Control led the public health response. The Nigeria Ministry of Defence Health Implementation Programme (MODHIP), in partnership with the US Army Medical Research Directorate – Africa/Nigeria, responded immediately to the pandemic by establishing a public health emergency operations center to coordinate the military response in support of national efforts. MODHIP has 5 functional units and 6 pillars that coordinate testing, surveillance, case management, risk communication, logistics, research, and infection prevention and control. It developed an incident action plan and each pillar had its own terms of reference to guide specific response activities while preventing duplication of efforts within the military and the Nigeria Centre for Disease Control. In addition, awareness and sensitization sessions were conducted on preventive practices for COVID-19 and infrastructure was provided for hand hygiene and screening at all military facilities. Military laboratories were configured for SARS-CoV-2 testing while selected military health facilities were equipped and designated as COVID-19 treatment centers. Research proposals aimed at better understanding the disease and controlling it were also developed. The traditional combat role of the military was redirected to complement this public health emergency response. In this article, we highlight gaps, opportunities, and lessons to improve military participation in public health emergency response in the future. More funding and multisectoral collaboration with civilian institutions are key to strengthening military public health emergency preparedness and response capabilities.

## Background

In December 2019, Chinese authorities alerted the World Health Organization (WHO) of a pneumonia of unknown origin in a wholesale seafood market in Wuhan, China.^1^ It was officially named COVID-19 in February 2020.^2^ The virus spread rapidly from Asia to the rest of the world, affecting hundreds of millions of people, killing millions of people, and devastating the global economy in its wake.^3^ As of May 4, 2022, over 515 million COVID-19 cases and over 6.2 million deaths have been reported worldwide.^4^ The first confirmed case of COVID-19 in Nigeria was an Italian national who arrived from Milan, Italy, via the Murtala Mohammed International Airport in Lagos on February 27, 2020.^5^ In Nigeria, as of April 17, 2022, 255,674 cases had been confirmed, with 3,143 deaths reported across all 36 states and the Federal Capital Territory; Lagos State and the Federal Capital Territory accounted for about 50% of all cases.^6^

After the Nigeria Centre for Disease Control (NCDC) activated a COVID-19 emergency operations centre (EOC) on February 27, 2020, the federal government of Nigeria launched the Presidential Task Force on COVID-19 on March 9, 2020, with an overarching mandate to coordinate and oversee the country's multisectoral and intergovernmental response to the pandemic.^7^ The chief of defense for training and operations was the official representative of the Nigerian military in the task force. He also chaired the Armed Forces Committee on COVID-19, which was established to oversee the response of the military.

Previously considered a last resort, militaries have been gaining prominence as key contributors in response efforts to humanitarian crises, health emergencies, and epidemic/pandemic preparedness. Historically, military personnel have been disproportionately affected by infectious disease outbreaks in both peacetime and war,^8-10^ sometimes serving as conduits of spread across continents.^9-11^ This spread is facilitated by movement of troops within and across national borders, exposure to disease agents, and a combination of physical, environmental, social, and psychological factors that can suppress the immune defenses of immunologically naive soldiers.^11-13^ Such infectious diseases have been shown to have a substantial impact on militaries, not only in terms of mortality and morbidity but also in operational readiness.^10^

Coupled with the recent emergence of the Global Health Security Agenda and its strong emphasis on security, defense, and law enforcement, this longstanding association between the military and diseases of public health concern has led to a codependency between military and civilian health institutions.^13,14^ Militaries have thus played leading roles in the discovery of lifesaving therapeutics and technologies while building tremendous in-house medical and health capacity and resources.^10,11^ Recognition of the military as a previously untapped resource has led to their increasing involvement in infectious disease outbreak response efforts, as highlighted by the multiple deployments of local and international soldiers to manage the 2014-2016 Ebola outbreak in West Africa.^14-19^

Almost every country has adopted a whole-of-nation approach in responding to the COVID-19 pandemic by mobilizing the military using varied organizational structures and activities, such as minimal technical military support, blended civil–military responses, and military-led responses.^14^ The scope of direct military involvement has extended beyond the health sector and broadly includes law enforcement and border control, social welfare and humanitarian assistance, and logistics. Specific activities have included lockdown enforcement, border management, food distribution, support for nursing homes, use of military hospitals for civilians, use of military personnel to augment staff in civilian hospitals, building of temporary field hospitals, provision of psychological support, testing and laboratory support, contact tracing, infodemic management, disinfection of public spaces, repatriation and evacuation of citizens, defense industry production of essential personal protective equipment (PPE), procurement and transport of medical supplies, focused research and development of technology addressing the pandemic, and vaccine development and delivery.^19-27^

To ensure an all-encompassing response, the Nigerian military leveraged its preexisting relationship with the NCDC, the agency mandated by law to coordinate Nigeria's response to disease outbreaks. The military's relationship with the NCDC had existed for about 5 years before the pandemic and focused on disease surveillance and provision of security for public health personnel in security-challenged areas.

The Ministry of Defence Health Implementation Programme (MODHIP) is a military partnership with the US Department of Defense through the US Army Medical Research Directorate–Africa/Nigeria (USAMRD-A/N) that interfaces with the NCDC. The program was established in 2005 to control the spread of HIV/AIDS within the armed forces of Nigeria, but its mandate has since expanded to include other emerging and reemerging diseases of military and public health significance. MODHIP supports over 40 military health service delivery facilities across the country.

During the period under review (March 2020 to April 2021), MODHIP was able to coordinate the response within the arms of the military (ie, Nigerian Army, Nigerian Navy, Nigerian Air Force) to prevent duplication of efforts and to support the national public health response. In this article, we describe the Nigerian military's role in supporting the ongoing national COVID-19 pandemic response and highlight specific activities.

## The Military COVID-19 Public Health Emergency Operations Centre

On March 20, 2020, MODHIP established and activated a new COVID-19 public health emergency operations center (PHEOC) at level 3, the highest level of response, which requires national coordination and the use of all available resources. About 6 months before the COVID-19 outbreak in China, 13 military officers were trained as public health emergency managers by a team from the NCDC, US Centers for Disease Control and Prevention (US CDC) Nigeria office, and the US CDC in Atlanta, as part of Nigeria's preparedness for disease outbreaks. These officers formed the core of the military pandemic response in Nigeria. PHEOC membership was made up of subject matter experts drawn from MODHIP, the Office of the National Security Adviser, and the NCDC. During the activation of PHEOC, a US CDC Nigeria office representative conducted an orientation workshop on operationalizing an incident management system for the team.

**Figure 1.**
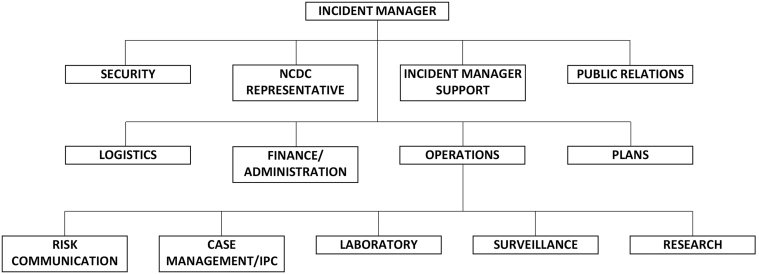
Ministry of Defence Health Implementation Programme Public Health Emergency Operations Center organizational chart. Abbreviations: IPC, infection prevention and control; NCDC, Nigeria Centre for Disease Control.

MODHIP adapted the WHO incident management system model^28^ to operate PHEOC and coordinate the response, as this was the first time such a structure was being implemented in response to a public health emergency within the Nigerian military. The incident management system structure had 5 main functional units: management, operations, logistics, plans, and finance/administration. The operations section was further subdivided into 6 pillars: laboratory, case management, surveillance, risk communication, research, and infection prevention and control (IPC). Each pillar had its own terms of reference with expected deliverables. Figure 1 shows the structure of the COVID-19 PHEOC incident management system. PHEOC held weekly virtual meetings in accordance with the NCDC's physical distancing protocols to review the preceding week's activities and offer solutions. Similarly, weekly virtual meetings were held with all treatment facility commanders and the PHEOC case management pillar.

## PHEOC Response Activities

### Risk Communication

Following the detection of COVID-19 in Nigeria, MODHIP disseminated information, education, and communication materials to all 40 program-supported sites. The NCDC risk communication team trained military personnel across the country on effective community engagement, health education, rumor management, behavior change, and health promotion. COVID-19 awareness and sensitization measures were instituted in military barracks, with a special focus on physical distancing, regular handwashing, use of alcohol-based hand sanitizers, wearing of facemasks, and periodic decontamination of offices. MODHIP conducted a rapid communication needs assessment in conjunction with active participation and engagement of barrack community organizations such as the barrack health club, churches, mosques, and military commanders in Mogadishu Cantonment, Abuja, to identify COVID-19 communication gaps and determine the most effective channels for sharing information. MODHIP used the findings to develop a military risk communication strategy for COVID-19.

### Infection Prevention and Control

PHEOC instituted IPC measures to curb the spread of COVID-19 across most military formations. These measures involved administrative and engineering controls in addition to using PPE. Following WHO recommendations on universal masking, Nigerian Defence Headquarters ordered all personnel, civilian residents, and visitors entering military formations to wear facemasks and perform hand hygiene at entry points. Additional measures included physical distancing directives instituted by local commanders. To enforce physical distancing, all ongoing military training programs were suspended and all parades were limited to 25 soldiers at a time. Soldiers working in offices where physical distancing could not be enforced were scheduled for shift duties to prevent overcrowding. Facility commanders ensured that tents were pitched to increase space capacity and maintain physical distancing in waiting areas. As part of response activities, MODHIP provided PPE to all supported sites and trained over 400 healthcare workers on IPC measures. The lead for the EOC IPC pillar was appointed a member of the NCDC COVID-19 EOC IPC team and attended meetings, participated in decisionmaking, and facilitated information sharing between both EOCs. The team also supported the establishment of committees at the facility level to ensure adherence to IPC measures and rational use of PPE.

### Surveillance

**Figure 2.**
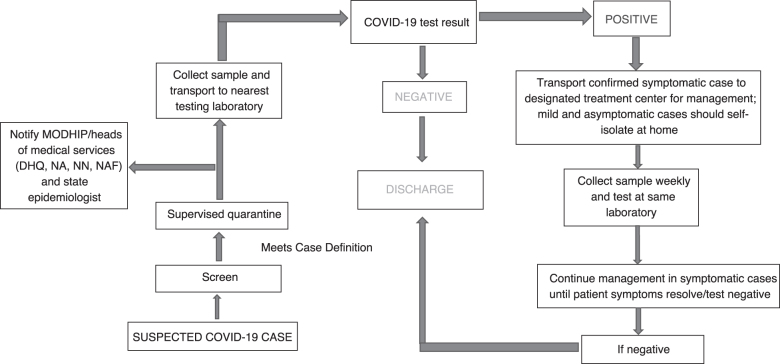
Flow chart for COVID-19 case detection and management in military treatment centers. Abbreviations: DHQ, Defence Headquarters; MODHIP, Ministry of Defence Health Implementation Programme; NA, Nigerian Army; NAF, Nigerian Air Force; NN, Nigerian Navy.

MODHIP's approach to surveillance was based on the principle of screen, isolate, and notify, and was instituted at the entrances of all military facilities. Temperature checks were conducted using infrared thermometers and persons with temperature above 38°C were safely guided to the barrack health facility for further examination and possible sample collection if they met the case definition for COVID-19; all sites received the NCDC case definitions for COVID-19 and data collection tools. Data entry clerks uploaded all data from MODHIP's laboratories and sample collection sites to the national real-time surveillance database known as SORMAS (Surveillance Outbreak Response Management and Analysis System). Joint surveillance teams consisting of military and state health officials conducted active case search and contact tracing for suspected COVID-19 infected persons within affected facilities, barracks, and bases. The case identification and management flowchart is shown in Figure 2.

### Laboratory

**Figure 3.**
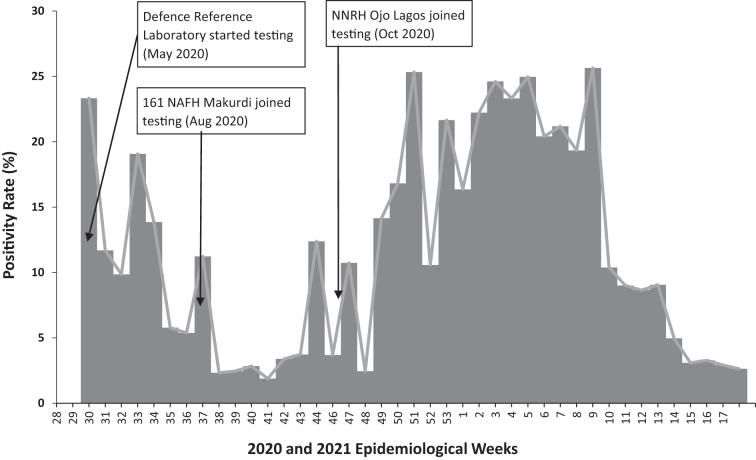
COVID-19 positivity rate from Nigerian military laboratories, May 2020 to April 2021. Abbreviations: NAFH, Nigerian Air Force Hospital Laboratory; NNRH, Nigerian Navy Reference Hospital Laboratory.

To test for SARS-CoV-2, MODHIP used existing laboratory equipment (eg, cobas 8800, GeneXpert, LightCycler 480) that was acquired with support from the US President's Emergency Fund for AIDS Relief (PEPFAR) for HIV/AIDS diagnostic services. Between May and October 2020, MODHIP configured 3 military laboratories and integrated them into the NCDC national COVID-19 laboratory network (Figure 3), with 2 more facilities awaiting activation and integration. The Defence Reference Laboratory in Abuja uses cobas 8800 (Roche Diagnostics, Basel, Switzerland) and Applied Biosystems 7500 (Thermo Fisher Scientific, Waltham, MA) as testing platforms, whereas the Nigerian Air Force Hospital Laboratory and Nigerian Navy Reference Hospital Laboratory used LightCycler 480 (Roche Diagnostics, Basel, Switzerland) and GeneXpert (Cepheid, Sunnyvale, CA). Laboratory staff were rotated in 2 shifts to achieve physical distancing. Facilitators from MODHIP, NCDC, and the Nigerian Biological Safety Association trained military laboratory personnel on IPC, biosafety/biosecurity, sample collection, and sample analysis for SARS-CoV-2. Laboratory reagents, test kits, consumables, and PPE were provided by MODHIP, in conjunction with NCDC, while the National Tuberculosis and Leprosy Control Programme provided support for the GeneXpert test kits. In addition to testing military personnel and their families, the laboratories also supported COVID-19 testing in 10 (out of 36) states of Nigeria.

The laboratories are located in various parts of Nigeria and maintain open communication with the NCDC. Test results generated by the military laboratories are fed into SORMAS through their respective state PHEOCs, except the Defence Reference Laboratory, which reports directly to SORMAS. MODHIP also established sample collection sites in strategic military locations across the country for military personnel, their families, and contiguous civilian communities. COVID-19 testing performances during the period under review from the 3 laboratories are shown in Figure 3.

### Case Management

In April 2020, the NCDC, with financial resources and military aircraft provided by the Armed Forces of Nigeria Committee on COVID-19, trained military multidisciplinary rapid response teams to complement the NCDC-led national efforts. The committee then deployed the teams to high-burden COVID-19 states within each of the 6 geopolitical zones in the country. Each rapid response team consisted of physicians, nurses, laboratory scientists, pharmacists, and data/health information managers. The lead for each team was tasked to support the state PHEOCs where they were deployed. With the support of MODHIP, the Nigerian Armed Forces Committee on COVID-19 also established 6 COVID-19 isolation and treatment centers—1 in each of the 6 geopolitical zones. Facilitators from NCDC and MODHIP trained military healthcare providers on IPC and COVID-19 case management using the NCDC-approved case management protocol. The lead for the case management pillar of the MODHIP EOC was appointed a member of the NCDC COVID-19 EOC case management team. Case managers in military treatment centers were mentored remotely via Zoom teleconferencing from the EOC. The treatment centers currently provide services to both military and civilians clients. MODHIP furnished all 6 treatment centers with necessary consumables and equipment for COVID-19 patient management.

### Logistics

The logistics pillar coordinated the process of procuring and delivering supplies and equipment required to upgrade treatment centers and ancillary units, including PPE, ICU beds, intensive care monitors, ventilators, patient management documents, medical consumables, hand sanitizers, and other decontamination agents. During the national lockdown, MODHIP purchased medical consumables from accredited local vendors and ensured their seamless delivery to service delivery sites through the use of military aircrafts provided by the Nigerian Air Force. The aircrafts were also instrumental in moving military rapid response teams across the country, as well as supporting civilian public health authorities with conveyance of national strategic-level response coordinators and other civilian healthcare personnel during the lockdown phase. The Nigerian Air Force also deployed an air ambulance to transport some critically ill patients to COVID-19 treatment centers.

### Research

The research pillar was tasked by the EOC to forecast the likely course of the pandemic response, use data from the field to guide the ongoing response, and provide evidence to inform future outbreak response activities. The EOC also mandated the pillar to access study grants or other COVID-19 research-related funding, engage in research studies, and disseminate their findings to the scientific community. The research pillar developed and submitted research proposals for antigen-based SARS-CoV-2 rapid test kit validation and COVID-19 seroprevalence studies to the NCDC for consideration. They also supported the IPC pillar to develop a protocol aimed at determining COVID-19 knowledge, attitudes, and practice among health workers (doctors, nurses, phlebotomists, records staff, HIV counselors, cleaning attendants) in 4 military health facilities, with a view toward identifying gaps that need remediation. The protocol also included a demonstration that assessed the effectiveness of a bundle of IPC interventions (eg, hand hygiene, appropriate use of PPE, maintenance of physical distancing protocols) in reducing SARS-CoV-2 infection among health workers in the 4 PEPFAR-supported program sites. The process of documenting our findings is ongoing.

In September 2020, MODHIP's Clinical Research Center, which is affiliated with PHEOC, was selected as a Tier 1 site of the Africa CDC Consortium for COVID-19 Vaccine Clinical Trials. In collaboration with the African Center of Excellence for Genomics of Infectious Diseases, the Clinical Research Center submitted a grant application in April 2021 to study the effect of SARS-CoV2 on vaccine-induced and naturally acquired immune responses in Nigeria, in response to an Africa CDC call for proposals. The grant was approved and is in the process of being implemented. The Clinical Research Center is also collaborating with Sanofi Pasteur to implement a Phase III vaccine trial to determine the immunogenicity, efficacy, and safety of the Sanofi Pasteur COVID-19 vaccine within the Nigerian population prior to its introduction in the country.

### Coordination

PHEOC coordinated the overall response from the MODHIP liaison office. The director general of MODHIP and the country director of USAMRD-A/N both served as incident managers, ensuring efficient 2-way flow of information between all stakeholders and efficient use of resources. The MODHIP director general coordinated response activities with military authorities, the Office of the National Security Adviser, and the NCDC, while the USAMRD-A/N country director was responsible for communicating with US government agencies and other international stakeholders.

The lead for the operations pillar assigned responsibility for the daily guidance and monitoring of the pandemic response activities at service delivery sites to the respective pillars who then provided progress reports at weekly meetings. The plans unit of the incident management system developed and deployed necessary documents such as standard operating procedures, protocols, and incident action plans for operational guidance. The operations unit of the incident management system linked program sites to civilian health authorities through the respective state epidemiologists for incorporation into the state pandemic response structure to avoid building a military structure that would be parallel to that of the NCDC.

## Discussion

In response to the emerging HIV/AIDS pandemic in the early 1990s, MODHIP developed robust diagnostic, preventive, and treatment capacities to achieve epidemic control within the Nigerian military. To this end, resources were provided by PEPFAR; the Global Fund to Fight HIV/AIDS, Tuberculosis and Malaria; and other international development agencies/partners. MODHIP leveraged these resources to coordinate laboratory-based disease surveillance using molecular techniques made available by these agencies to respond to the COVID-19 pandemic. Using these resources for the pandemic was a clear opportunity for MODHIP, which had also opened doors to explore the frontiers of genetic sequencing to detect existing and emerging infectious pathogens, especially in other low- and middle-income countries like Nigeria.

The unprecedented and innovative decision to establish and activate a military COVID-19 PHEOC in Nigeria that mirrors the existing national PHEOC is noteworthy. To the best of our knowledge, no similar dedicated military PHEOC exists elsewhere. It is important to note that this military PHEOC was not intended to be independent or parallel; rather, it was closely embedded within the national structure to ensure efficiency and avoid duplication of response efforts.

The establishment of a military PHEOC was deemed necessary for 4 major reasons. First, there is a well-established association between the military and infectious disease outbreaks, and the military was considered a likely vehicle for COVID-19 importation and onward local transmission. Second, the military is a distinct population that may be inaccessible to civilian response efforts. Third, PHEOC was considered an effective tool for aggregation and deployment of all military medical and nonmedical resources toward positive public health outcomes, given the largely parallel but dependent and centralized nature of the different arms of the military. Last, the hope was that the military, with its substantive and underexploited health resources, could provide surge capacity in the event of an overwhelmed civilian health system.

The extent to which the previously stated objectives were met during the March 2020 to April 2021 study period is mixed. The military does not appear to have been a hotspot or driver for the COVID-19 outbreak in Nigeria, which is likely due to a combination of factors, but the possible benefits of PHEOC cannot be ruled out. As documented earlier, numerous activities were carried out within military installations and barrack communities through the various PHEOC pillars, which would have been difficult to achieve through civilian efforts. PHEOC does not have command authority over the various military arms and their health services, and, therefore, MODHIP was responsible for obtaining voluntary buy-in and enthusiasm from the different military hierarchies to ensure effective coordination. This cooperation was suboptimal, however, especially in regard to case management efforts in some facilities. PHEOC provided effective oversight mainly for the 40 program-supported sites but had limited influence over other military facilities across Nigeria. As a result, effective monitoring of COVID-19 management protocols in most military facilities was not possible because data from these facilities were inaccessible. This challenge was not anticipated before the response. Successfully preparing for a future public health event will require developing a response framework that empowers PHEOC with necessary authority over all military facilities in public health emergency settings. It is our hope that lessons learned during the COVID-19 pandemic will improve cohesion in the future. Finally, the level of support required for a wider national response is substantial—especially in regard to laboratory testing facilities, logistics, and research. Unpublished data from MODHIP and NCDC show that military laboratories performed as much as 1.4% of the samples tested nationally, despite the military comprising only about 0.1% of Nigeria's population.^29,30^ Also commendable is PHEOC contributions to COVID-19 research and development. The potential contribution of the Clinical Research Center to COVID-19 vaccine efficacy and safety trials and improved understanding of the impact of SARS-CoV-2 variants on naturally acquired and vaccine-induced immune responses are considered groundbreaking in the Nigerian context.

Other key challenges also hampered response efforts. Of significance was the lack of a military public health emergency response plan, as well as the absence of dedicated preexisting emergency funds to facilitate the activities of the various EOC pillars. These challenges contributed to the 3-month delay between activation of PHEOC and operationalization of the first designated military COVID-19 treatment center. Despite the unavailability of dedicated public health emergency response funds, MODHIP was able to mobilize resources from the Ministry of Defence and the Presidential Task Force on COVID-19 while USAMRD-A/N provided support through diagnostics, PPE, and other medical consumables.

Another factor contributing to delayed implementation was the relatively new and vaguely defined collaboration and communication channels between MODHIP and the NCDC. This hampered prompt access to technical support needed at the initial stages of response, especially in the activation and integration of military testing laboratories into the national COVID-19 laboratory network. We recommend strengthening this collaboration by establishing an appropriate engagement framework between the 2 institutions before the next outbreak. Collaborations between militaries and civilian organizations at various levels are important in the prevention and control of infectious diseases and are best performed before the onset of an outbreak or epidemic.^10^

A final challenge that may have affected the response was PHEOC's narrow scope of responsibilities. In addition to core public health disciplines, other relevant sectors should also be included; for example, the engineering corps of other militaries worldwide play a prominent role in supporting the quick setup of temporary field hospitals for enhanced case management capacity.^31^ Future response efforts should also explore the possibility of including military religious leaders to provide community engagement, risk communication, infodemic management, and psychosocial support for affected troops and their families. These nonmedical stakeholders are critical to a successful response and their lack of involvement in PHEOC was a key lesson learned in this first attempt at implementing the incident management system structure in military public health response.

## Conclusion

The role of military health systems in population health is poorly understood. In this article, we describe the structure and functional response of the Nigerian military during the COVID-19 pandemic. We underscore the important roles the military can play in public health emergency preparedness and response in support of the national effort. In addition, we highlight the need for multisectoral and multinational collaborations in pandemic response. The COVID-19 pandemic has presented a landmark moment in defining the roles of militaries in public health. Militaries offer strengthened human resources, a rigid command structure, and immense logistic capabilities. In addition, they are deployable at short notice, a vital factor in responding to an emerging health crisis. The attempt to draw on this largely untapped resource by setting up a military PHEOC in Nigeria has been commendable. The Nigeria military response presents a model that can be improved upon and sustained as a readily available rapid response option in managing future public health threats. In addition to demonstrable achievements, the experience has also revealed gaps and challenges. Next steps required to strengthen this novel idea include developing a multidisciplinary military public health response plan, further strengthening capacity within the military, engaging with key stakeholders in the armed forces to ensure buy-in and ownership, making emergency funds available at all times, adopting a much wider multisectoral representation, and strengthening linkages with civilian institutions.

## References

[1] World Health Organization. COVID-19 - China. Published January 5, 2020. Accessed May 1, 2021. https://www.who.int/csr/don/05-january-2020-pneumonia-of-unkown-cause-china/en/

[2] World Health Organization (WHO). WHO Director-General's remarks at the media briefing on 2019-nCoV on 11 February 2020. Published February 11, 2020. Accessed May 1, 2021. https://www.who.int/director-general/speeches/detail/who-director-general-s-remarks-at-the-media-briefing-on-2019-ncov-on-11-february-2020

[3] Jackson JK, Weiss MA, Schwarzenberg AB, Nelson RM, Sutter KM, Sutherland MD. Global Economic Effects of COVID-19 (R46270). Version 82. Washington, DC: Congressional Research Service; 2021. https://crsreports.congress.gov/product/details?prodcode=R46270

[4] Johns Hopkins Coronavirus Resource Center. COVID-19 dashboard by the Center for Systems Science and Engineering (CSSE) at Johns Hopkins University (JHU). Accessed May 4, 2022. https://coronavirus.jhu.edu/map.html

[5] Nigeria Centre for Disease Control. COVID-19 outbreak in Nigeria: situation report no. 001. Published February 29, 2020. Accessed May 4, 2022. https://ncdc.gov.ng/diseases/sitreps/?cat=14&name=An update of COVID-19 outbreak in Nigeria

[6] Nigeria Centre for Disease Control (NCDC ). COVID-19 situation report: weekly epidemiological report 79. Published April 17, 2022. Accessed May 4, 2022. https://ncdc.gov.ng/diseases/sitreps/?cat=14&name=An update of COVID-19 outbreak in Nigeria

[7] Dan-Nwafor C, Ochu CL, Elimian K, et al. Nigeria's public health response to the COVID-19 pandemic: January to May 2020. J Glob Health. 2020;10(2):020399. 33274062 10.7189/jogh.10.020399 PMC7696244

[8] Connolly MA, Heymann DL. Deadly comrades: war and infectious diseases. Lancet. 2002;360(suppl):S23-S24. 12504490 10.1016/s0140-6736(02)11807-1

[9] Byerly CR. The U.S. military and the influenza pandemic of 1918-1919. Public Health Rep. 2010;125(suppl 3):82-91. 20568570 PMC2862337

[10] Ho ZJ, Hwang YF, Lee JM. Emerging and re-emerging infectious diseases: challenges and opportunities for militaries. Mil Med Res. 2014;1(21):1-10. 25722877 10.1186/2054-9369-1-21 PMC4341224

[11] Chretien JP, Blazes DL, Coldren RL, et al. The importance of militaries from developing countries in global infectious disease surveillance. Bull World Health Organ. 2007;85(3):174-180. 17486207 10.2471/BLT.06.037101 PMC2636235

[12] Murray CK, Horvath LL. An approach to prevention of infectious diseases during military deployments. Clin Infect Dis. 2007;44(3):424-430. 17205453 10.1086/510680

[13] Dutton LK, Rhee PC, Shin AY, Ehrlichman RJ, Shemin RJ. Combating an invisible enemy: the American military response to global pandemics. Mil Med Res. 2021;8(1):8. Published correction appears in Mil Med Res. 2021;8(1):11. 33487173 10.1186/s40779-021-00299-3 PMC7829065

[14] Gibson-Fall F. Military responses to COVID-19, emerging trends in global civil-military engagements. Rev Int Stud. 2021;47(2):155-170.

[15] Kaplan J, Easton-Calabria E. Military medical innovation and the Ebola response: a unique space for humanitarian civil-military engagement. Humanit Exch. 2015;64:7-9.

[16] Ma H, Dong J-P, Zhou N, Pu W. Military–civilian cooperative emergency response to infectious disease prevention and control in China. Mil Med Res. 2016;3:39. 28050261 10.1186/s40779-016-0109-y PMC5203723

[17] Hammer A. Military humanitarian intervention: a new war on disease? J Biosecur Biosaf Biodef Law. 2017;8(1):111-128.

[18] Chatham House Centre on Global Health Security. The Next Ebola: Considering the Role of the Military in Future Epidemic Response. London: Chatham House; 2017. Accessed March 12, 2022. https://www.chathamhouse.org/sites/default/files/events/2017-03-31-next-ebola-role-of-military-meeting-summary.pdf

[19] Kalkman JP. Military crisis responses to COVID-19. J Contingencies Crisis Manag. September 27, 2020. doi:10.1111/1468-5973.12328 PMC7537208 40477147

[20] Correll DS. Senegal, Ghana employ medical training from U.S. military to respond to COVID-19. Military Times. April 7, 2020. Accessed January 30, 2022. https://www.militarytimes.com/news/coronavirus/2020/04/07/senegal-ghana-employ-medical-training-from-us-military-to-respond-to-covid-19/

[21] Solomon S. As militaries enforce coronavirus quarantine, experts warn of escalating violence. VOA. April 24, 2020. Accessed February 2, 2022. https://www.voanews.com/a/covid-19-pandemic_militaries-enforce-coronavirus-quarantine-experts-warn-escalating-violence/6188151.html

[22] Kazibwe J, Gad M, Gheorghe A, Bricknell M. Using military health systems in the response to COVID-19. Center for Global Development. Published June 15, 2020. Accessed January 1, 2022. https://www.cgdev.org/blog/using-military-health-systems-response-covid-19

[23] Laţici T. The role of armed forces in the fight against coronavirus. European Parliament. Published April 28, 2020. Accessed March 14, 2022. https://www.europarl.europa.eu/thinktank/en/document/EPRS_BRI(2020)649401

[24] US Department of Defense. Coronavirus: DOD response timeline. Updated March 17, 2022. Accessed March 23, 2022. https://www.defense.gov/Spotlights/Coronavirus-DOD-Response/Timeline/

[25] Forces Net. COVID: how the military's been involved in fighting coronavirus. Published February 18, 2021. Accessed March 23, 2022. https://www.forces.net/news/coronavirus-how-military-helping

[26] Government of Canada. Military response to COVID-19. 2020. Accessed January 2, 2022. https://www.canada.ca/en/department-national-defence/campaigns/covid-19-military-response.html

[27] Gad M, Kazibwe J, Quirk E, Gheorghe A, Homan Z, Bricknell M. Civil-military cooperation in the early response to the COVID-19 pandemic in six European countries. BMJ Mil Health. 2021;167(4):234-243. 10.1136/bmjmilitary-2020-001721 PMC8011427 33785587

[28] World Health Organization (WHO). Framework for a Public Health Emergency Operations Centre, 2015. Geneva: WHO; 2015. Accessed March 14, 2022. https://apps.who.int/iris/bitstream/handle/10665/196135/9789241565134_eng.pdf?sequence=1

[29] The World Bank. Armed forces personnel, total - Nigeria. Accessed June 24, 2021. https://data.worldbank.org/indicator/MS.MIL.TOTL.P1?locations=NG

[30] The World Bank. Population, total - Nigeria. Accessed June 24, 2021. https://data.worldbank.org/indicator/SP.POP.TOTL?locations=NG

[31] Cronk TM. Corps of Engineers takes on 28 COVID-19 bed facilities. DOD News. Published April 17, 2020. Accessed February 2, 2022. https://www.defense.gov/News/News-Stories/Article/Article/2154305/corps-of-engineers-takes-on-28-covid-19-bed-facilities/

